# Binding of a viral IRES to the 40S subunit occurs in two successive steps mediated by eS25

**DOI:** 10.1093/nar/gkaa547

**Published:** 2020-07-01

**Authors:** Beth Walters, Armend Axhemi, Eckhard Jankowsky, Sunnie R Thompson

**Affiliations:** Department of Microbiology, University of Alabama at Birmingham, Birmingham, AL 35294, USA; Center for RNA Science and Therapeutics, Case Western Reserve University, Cleveland, OH 44106, USA; Center for RNA Science and Therapeutics, Case Western Reserve University, Cleveland, OH 44106, USA; Department of Microbiology, University of Alabama at Birmingham, Birmingham, AL 35294, USA

## Abstract

The mechanism for how internal ribosome entry sites (IRESs) recruit ribosomes to initiate translation of an mRNA is not completely understood. We investigated how a 40S subunit was recruited by the cricket paralysis virus intergenic region (CrPV IGR) IRES to form a stable 40S–IRES complex. Kinetic binding studies revealed that formation of the complex between the CrPV IGR and the 40S subunit consisted of two-steps: an initial fast binding step of the IRES to the 40S ribosomal subunit, followed by a slow unimolecular reaction consistent with a conformational change that stabilized the complex. We further showed that the ribosomal protein S25 (eS25), which is required by functionally and structurally diverse IRESs, impacts both steps of the complex formation. Mutations in eS25 that reduced CrPV IGR IRES activity either decreased 40S–IRES complex formation, or increased the rate of the conformational change that was required to form a stable 40S–IRES complex. Our data are consistent with a model in which eS25 facilitates initial binding of the CrPV IGR IRES to the 40S while ensuring that the conformational change stabilizing the 40S–IRES complex does not occur prematurely.

## INTRODUCTION

Canonical cap-dependent initiation in eukaryotes requires a 5′ cap structure (^m7^GpppN) on the mRNA, which is recognized by eukaryotic initiation factors that function to bring the 40S subunit to the 5′end of the mRNA where upon it scans down the mRNA until it reaches the start codon where 60S joining occurs (1). However, many positive stranded RNA viruses that depend on the cellular translation machinery for protein synthesis, as well as some cellular mRNAs, use a cap-independent mechanism of translation initiation whereby an RNA element, termed an internal ribosome entry site (IRES), recruits the ribosomes internally to initiate protein synthesis. We have shown that structurally and functionally diverse IRESs rely on the ribosomal protein S25 (eS25/RPS25) (2). In order to better understand the role of eS25 in IRES-mediated initiation we have investigated its role in 40S recruitment of a model viral IRES, the intergenic region (IGR) cricket paralysis virus (CrPV) IRES, from the family of *Dicistroviridae*.

The CrPV IGR IRES is 190 nucleotides and forms a compact RNA structure with three pseudoknots (PKI, II and III) (3,4). The CrPV IGR IRES can bind to 40S subunits and form 80S complexes in the absence of any initiation factors (5). PKII and PKIII of the CrPV IGR IRES form a compact core with SL2.1 (SLIV) and 2.3 (SLV) interacting with the 40S ribosome (6–9). PKI mimics an anticodon stem loop of a tRNA recognizing an mRNA codon (10) and is positioned into the decoding center (A-site) initially. Rather than initiating at an AUG start codon in the peptidyl (P)-site, PKI, which establishes the reading frame (11) is translocated to the P-site for initiation of an alanine codon the A-site of the ribosome (5,8,12–14).

CrPV IGR IRES binding to the 40S ribosomal subunits is dependent on eS25 (15). The IGR IRES binding to the 40S subunit induces a conformational change in the 40S subunit (6,7) that is similar to the one induced by the hepatitis C viral IRES (16). Upon binding to the 40S subunit, the CrPV IGR IRES is in a compact conformation (6,7,9) and SL2.3 interacts with eS25 (8,15). Initially, the IRES occupies all three tRNA binding sites, exit (E-), P- and A-sites, on the ribosome (8). This initiation complex mimics an elongation ribosomal conformation in the pre-translocation state rather than an initiating ribosome (8). Addition of an aminoacyl tRNA (aa-tRNA) and elongation factors, eEF2 and eEF1A, to the IRES–80S complex results in movement of PKI to the P-site bringing the first codon to be decoded into the A-site (17). Once the codon in the A-site is decoded, a second slower translocation event occurs placing the decoded codon into the P-site and PKI becomes disrupted as it moves into the E-site (9). Binding, and the translocation of the IRES through the ribosome, is a dynamic process whereby the IRES conformation changes as does its contacts with eS25 (9). The movement of tRNAs and mRNA relative to the 40S subunit requires eEF2 and rotation of the 40S head domain in order to unlock the steric barrier between the P- and E-sites.

In order to better understand how IRESs recruit and manipulate ribosomes to initiate translation, we performed kinetic binding studies. Our data show that recruitment of the CrPV IGR IRES to the 40S subunit occurred in two consecutive steps: fast initial binding of the IRES to the 40S followed by a slow unimolecular reaction consistent with a conformational change that stabilized the complex. Based on our previous finding that eS25 was required for 40S–IRES complex formation (15), we performed an extensive mutational analysis of the conserved eS25 residues and determined the effects of these mutations on IRES activity and on the steps leading to stable 40S–IRES complex formation. Mutations throughout eS25 affected IRES activity and multiple steps in 40S–IRES complex formation. Several eS25 mutations induced a 40S conformation that was unfavorable for initial CrPV IGR IRES binding, while others either decreased the rate of initial complex formation or increased the rate of the conformational change that stabilized complex formation. A faster conformational change correlated with a lower IRES activity, suggesting that the slow conformational change is important for forming a stable complex. Collectively, our findings indicate that CrPV IGR IRES recruitment of the 40S subunit is dependent on eS25 and occurs through a two-step process in which the timing of these steps is critical for IRES activity *in vivo*.

## MATERIALS AND METHODS

### Yeast strains and cell culture

*Saccharomyces cerevisiae* strains used in this study were: wild-type (BY4741: *MAT****α****his3Δ1 leu2Δ0 met15Δ0 ura3Δ0*), *rps25aΔbΔ* (ySRT221: *MAT****α****his3Δ1 leu2Δ0 lys2Δ0 ura3Δ0 rps25a*::KanMX *rps25b*::KanMX). Standard methods were used to grow and transform yeast strains (18,19). For all experiments yeast were grown at 30°C to mid-log phase in synthetic galactose (SG) medium for expression of eS25 from the plasmid.

### Generation of eS25 mutant plasmids

Single point mutations were made in the yeast pS25A rescue plasmid (2μ p*URA3*, Open Biosystems, catalog no. YSC3869–9518490) using a two-stage site directed mutagenesis PCR method (20). In brief, primers (Supplementary Table S1) specific for each mutation were generated using the PrimerX program (http://www.bioinformatics.org/primerx/) and individual PCR reactions were set up for each primer (200ng DNA template, 10pmol sense/antisense primer, 10× PFU buffer, 2.5 mM dNTPs, 2.5U *Pfu* DNA polymerase, 0.1U dUTPase). Reactions were combined with the addition of 0.05 U/μl *Pfu* DNA polymerase and 0.002 U/μl dUTPase, and the second PCR amplification was performed. Products were digested with DpnI (5U/μl) at 37°C for ≥2 h, and transformed into DH5α *Escherichia coli*. Mutations were confirmed by sequencing.

### Luciferase and β-galactosidase assays

One OD_600_ (approximately 3X10^7^) yeast cells at mid-log phase expressing either wild-type or mutant eS25 from a plasmid were pelleted and lysed in 100 μl 1× passive lysis buffer (PLB; Promega). Samples were vortexed for 15 s and incubated at 25°C for 105 s. 4 μl of lysate was assayed using the Dual Luciferase assay kit (Promega), following the manufacturer's protocol, with a Lumat LB 9507 luminometer (Berthold). All assays were performed in duplicate for *n* = 3 biological repeats.

### Ribosome isolation

Eight liters of yeast cells were pelleted for 10 mins at 4200 × g, 4°C, washed with 10 ml ddH_2_O. Pellets were re-suspended in 1× Ribo Buffer A (10 mM HEPES KOH, pH 7.4, 100 mM KOAc, pH 7.6, 2.5 mM Mg(OAc)_2_) with 2 mM DTT and frozen, dropwise in liquid N_2_. Yeast was lysed using the Spex Sample Prep Freezer/Mill 6870 (Metuchen, NJ, USA) and resuspended in 3 ml of Ribo Lysis Buffer (1× Ribo Buffer A, 2 mM DTT, 0.5 mM 4-benzenesulfonyl fluoride hydrochloride (AEBSF), 1× complete protease inhibitor EDTA-free (Roche)) per mg of pellet. Polysomes were pelleted from lysate at 6800 × g for 40 min, 4°C in a JS-5.3 Beckmann rotor, supernatant was layered onto a sucrose cushion (1× Ribo Buffer A, 500 mM KCl, 1 M sucrose, 2 mM DTT) and centrifuged in a Beckmann 70ti rotor at 290 000 × g for 106 min at 4°C. The polysome pellet was resuspended in 1.5 ml high salt wash (1× Ribo Buffer A, 500 mM KCl, 1 mg/ml heparin, 2 mM DTT) for 1 h at 4°C, layered over a sucrose cushion, and centrifuged in a Beckman TLA 110 rotor at 290 000 × g for 33 min at 4°C. The polysome pellet was resuspended in 1.5 ml subunit separation buffer (50 mM HEPES–KOH, pH 7.4, 500 mM KCl, 2 mM MgCl_2_, 2 mM DTT) plus 4 mM puromycin and incubated at 37°C for 45 min. The ribosomal subunits were separated by centrifugation through a 10–30% continuous sucrose gradient (50 mM HEPES–KOH, pH 7.4, 500 mM KCl, 5 mM MgCl_2_, 0.1 mM EDTA, 2 mM DTT) followed by fractionation measuring *A*_254_. Fractions containing the 40S and 60S subunits were concentrated in an Amicon Ultra-15 (Millipore) and the buffer was exchanged for subunit storage buffer (20 mM HEPES–KOH, pH 7.4, 100 mM KOAc, pH 7.6, 2.5 mM Mg(OAc)_2_, 250 mM sucrose, 2 mM DTT).

### Binding assays

α-^32^P-UTP radiolabeled CrPV IGR IRES run-off transcripts were generated using the T7 RiboMax large scale RNA production system (Promega) from the monocistronic luciferase plasmid (pSRT39) (5) linearized with NarI to generate a 261 nucleotide transcript containing nucleotides 6028–6213 of the CrPV virus, which consists of the entire IGR IRES plus the first 15 nucleotides of ORF2 followed by 40 nucleotides of the firefly luciferase ORF. The transcripts were gel-purified on a 6% denaturing polyacrylamide gel and eluted for 12 h in elution buffer (0.5 M NH_4_OAc, 1 mM EDTA, 0.1% SDS), acid phenol:chloroform (3:1) (Ambion) extracted, precipitated with 70% ethanol, and resuspended in RNase-free dH_2_O.

Filter binding assays were performed using constant amounts of radiolabeled CrPV IGR IRES RNA and 40S ribosomal subunits in 1x recon buffer (30 mM HEPES–KOH, pH 7.4, 100 mM KOAc, pH 7.6, 5 mM MgCl_2_, 2 mM DTT) with 15 μM cold non-competitor RNA *in vitro* transcribed from the pCDNA3.1 vector (Invitrogen) linearized with EcoRI. Complex formation occurred at 25°C. Reactions were passed over Whatman Protran BA 85 filters (Fisher) and washed with 1mL of 1× recon buffer and scintillation counted using an LS 6500 (Beckman). Competition assays were performed as above, except complexes were allowed to form for 20 min, then 450-fold molar excess cold competitor RNA (CrPV IGR IRES) was added over radiolabeled RNA. Time points were taken from 1 to 60 min after cold competitor RNA addition.

### Calculation of rate and equilibrium constants

**Figure 1.**
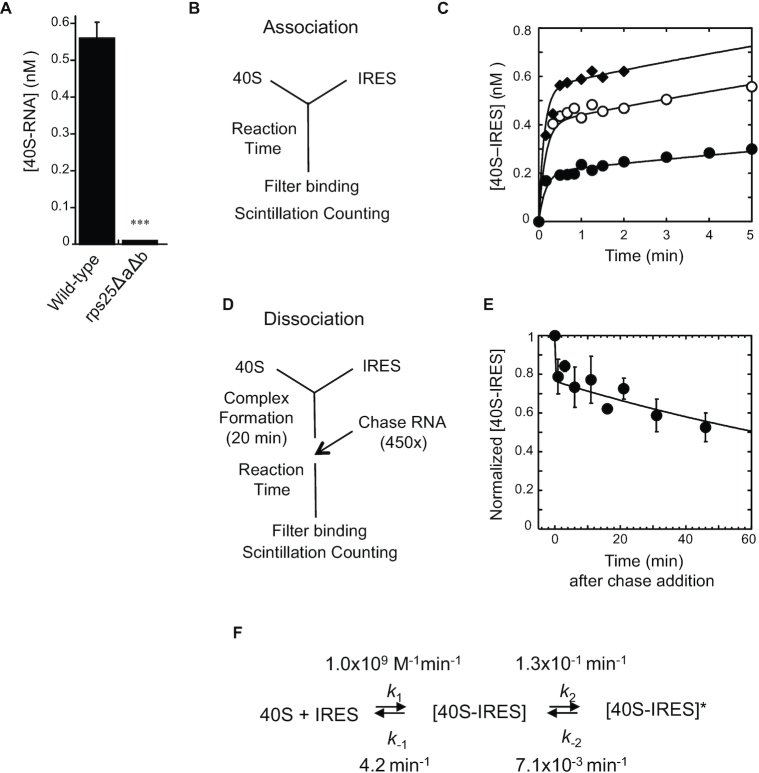
CrPV IGR IRES RNA binds to the 40S subunit in two reaction steps. (**A**) Binding of CrPV IGR IRES (4 nM) to the 40S subunit (1 nM) with and without eS25 (*rps25ΔaΔb*) was measured by filter binding assay after 3 min incubation. *n* = 3 biological repeats *P*-value indicated **≤ 0.01. *P*-value determined by unpaired *t*-test. (**B**) Reaction scheme for measuring 40S–IRES association at times ranging from 15 s to 5 min. (**C**) Filter binding assays for 40S–IRES complex formation were performed at various times (15 s to 5 min) with [40S] = 1 nM and the IRES concentration at 1 nM (•), 2.5 nM (○) or 4 nM (♦). Lines show the global fit to the kinetic model shown in panel (F). (**D**) Reaction scheme for how IRES dissociation from 40S was measured by chasing with unlabeled (cold) IRES RNA. Filter binding assays were performed at various times (ranging from 1 to 60 min) after the cold competitor CrPV IGR IRES (chase RNA) was added. (**E**) Time-course for measuring IRES dissociation from the complex that was formed using 2 nM 40S and 2 nM IRES for 20 min, then chased with 900 nM unlabeled CrPV IGR IRES RNA. Error bars indicate standard deviation for *n* ≥ 3 independent experiments. The line shows the global fit to the kinetic model shown in panel (F). (**F**) Reaction model and kinetic parameters for the two-step binding reaction for IRES binding to 40S subunits. ([40S–IRES]: intermediate complex; [40S–IRES]*: final complex; *k*_1_: association rate constant for step 1, *k*_–1_: dissociation rate constant for step 1, *k*_2_: forward rate constant for step 2; *k*_–2_: reverse rate constant for step 2).

Global fitting of association and dissociation time-courses was performed with *Kintek Global Kinetic Explorer* 6.0 (21,22). Data were fit to the reaction scheme described in the text (Figure 1F). To estimate initial parameters and sigma values for subsequent global fits, time courses were fit to a biphasic exponential function:

$$\begin{equation*}{\rm{frac }}\left[ {{\rm{bound}}} \right] = {{{a}}_1}\cdot{{\rm{e}}^{ - {{b}}1\cdot{\rm{t}}}} + {{{a}}_2}\cdot{{\rm{e}}^{ - {{b}}2\cdot{{t}}}} + {{c}}\end{equation*}$$

(frac [bound]: fraction of bound IRES RNA; *b*_1_, *b*_2_: observed rate constants for each phase; *a*_1_, *a*_2_: amplitudes for each phase; *c*: offset). The equilibrium between 40S_in_ (40S non-binding competent) and 40S_A_ (40S active) was set to favor complete 40S_A_ formation. Initial values for *k*_1_, *k*_-1_, *k*_2_ and *k*_–2_ were determined as follows: *k_1_ <* 1000 μM^−1^ s^−1^; *k*_2_ = *b*_2_ from association time courses. *k*_–1_ = *b*_1_ from the dissociation time course. *k*_–2_ = *b*_2_ from the dissociation time course. Initial parameters were further optimized with the *Dynamic Simulation* feature of *Kintek Explorer*, which allows variation of rate constants with continuous simulation. Next, association time-courses from at least three different IRES concentrations and dissociation time courses were globally fit with *k*_1_, *k*_–1_, *k*_2_, *k*_–2_ and *K’*_1/2_ set as variable. Obtained data were then used in several iterative rounds of global fitting until parameters no longer changed and a minimum *X*^2^ value was obtained for the global fit. For wild-type eS25 and each eS25 mutant, 70–100 individual data-points were used, respectively, to determine the four rate constants and the equilibrium value *K’*_1/2_ for the 40S_in_ to 40S_A_ transition for wild-type eS25 and each eS25 mutant. Parameters describing the quality for each of the global fits are listed in Supplementary Figure S1.

Free energy changes for the transition states were calculated according to the Eyring-Polanyi equation:

$$\begin{equation*}\Delta {{{G}}^\ddagger } = - {{RT}}\cdot{\rm{ln}}[\left( {k\cdot{{h}}} \right)\cdot{\left( {{{{k}}_{\rm{B}}}\cdot{{T}}} \right)^{ - 1}}]\end{equation*}$$

(*R*: gas constant; *T*: temperature (K), *k*: rate constant, *h*: Planck constant, *k*_B_: Boltzmann constant). To calculate the change in free energy for the transition state of step 1, which corresponds to *k*_1_, the second order association rate constant was converted to a pseudo-first order rate constant considering standard conditions (RNA concentrations set at [RNA] = 1 M).

The change in free energy for the equilibrium between 40S_in_ and 40S_A_ was calculated according to:

$$\begin{equation*}\Delta {{G}}^\circ = - {{RT}}\cdot{\rm{ln}}\left( {K{\text{'}_{1/2}}} \right).\end{equation*}$$

(*R*: gas constant; *T*: temperature (K), *K'*_1/2_: equilibrium constant between 40S_in_ and 40S_A_).

### Western analysis

50 ml yeast cultures were brought to 5% trichloroacetic acid (TCA), incubated on ice for 30 min and pelleted at 2800 × g for 4 min at 4°C. Pellets were washed 5 times with 4 ml 100% acetone and pelleted at 2800 × g for 4 min at 4°C. Pellets were dried on ice and stored at –80°C until resuspended in 300 μl (50 mM Tris–HCl, pH 7.5, 1 mM EDTA, 1% SDS) and 400 μl acid-washed glass beads (Sigma). Cells were lysed using a Retsch MM200 mixer mill for eight intervals of 1 min at a frequency of 30 (1/s). 8–10 μg of total protein was separated by 16% tricine SDS-PAGE, transferred to an Immobilon-FL polyvinylidene difluoridemembrane (Millipore Co., Milford, MA, USA), and probed with a 1° antibody (our rabbit polyclonal yeast RPS25, mouse monoclonal phosphoglycerate kinase (PGK) antibodies (#A6457, Invitrogen), or rabbit polyclonal uS6 (RPS6) antibody (#ab40820, AbCam)) then a 2° anti-rabbit or anti-mouse fluorochrome-conjugated antibody (#926–32212, Li-Cor, Lincoln, NE). Westerns were visualized and quantified using Odyssey scanner and Li-Cor software.

### Statistical analyses

A Student's *t*-test was used to determine significant differences in eS25 mutant IRES activity compared to wild-type eS25. An unpaired *t*-test was used to determine significance in binding activity (IRES to ribosome) of eS25 mutants compared to wild-type eS25 binding.

## RESULTS

### CrPV IGR IRES binding is a two-step reaction

Kinetic binding studies on the CrPV IGR IRES with purified 40S subunits with and without eS25 (*rps25ΔaΔb*) (Figure 1A) (15) revealed that the 40S–IRES association rate increased with increasing IRES concentrations (Figure 1B) indicating a bi-molecular binding reaction. However, the pronounced biphasic shape (Figure 1C), suggests multiple kinetically discrete steps or formation of multiple 40S–IRES complexes with distinct kinetic properties. The rate of IRES dissociation from the 40S subunit (Figure 1D) displayed a clear biphasic shape (Figure 1E) providing further support for the existence of multiple 40S–IRES complexes with distinct kinetic properties.

The simplest kinetic model to fit both association and dissociation time courses comprised two consecutive reversible steps (Figure 1F and Supplementary Figure S1). The data were not adequately described by simpler models with only one or two non-reversible steps, nor by models consisting of two parallel reversible reactions or two populations of 40S (Supplementary Figure S1). Rate constants for the kinetic model with two consecutive reversible steps were obtained by a global fit of all association and dissociation time courses (Figure 1F and Supplementary Table S2). The rate constants indicate a bimolecular binding reaction in which the IRES and the 40S form an initial 40S–IRES complex that converts in a unimolecular reaction to a second, distinct [40S–IRES]* complex. This unimolecular reaction step most likely represents a conformational change.

The initial binding step occurs with a rate constant close to the diffusion limit, while conversion to the [40S–IRES]* complex is slow, relative to the first bimolecular reaction step even above low nanomolar concentrations of reactants. The initial 40S–IRES complex is thermodynamically stable at low nanomolar IRES concentrations (*K’*_1/2_ = 4.2 nM). IRES dissociation from the intermediate state (*k*_–1_) is reflected in the first, fast phase of the dissociation time course (Figure 1E). The IRES is ∼30 times more likely to dissociate from this complex than to convert to [40S–IRES]*. Compared to the intermediate 40S–IRES complex, the [40S–IRES]* complex is long lived. Formation of the [40S–IRES]* complex is 18 times more likely than the back-reaction (*k*_–2_) (Figure 1F). The amplitude of the first phase of the dissociation time course roughly reflects the equilibrium between intermediate state, [40S–IRES], and second bound state, [40S–IRES]* (Figure 1E, F, and Supplementary Table S2). It is formally possible that the second step is essentially irreversible, in which case the rate constant *k*_-2_ would represent dissociation of the IRES from [40S–IRES]*. However, even in this scenario IRES dissociation from [40S–IRES]* is orders of magnitude slower than dissociation from the intermediate state, and none of the conclusions below would fundamentally change. Our results thus collectively indicate that IRES binding to the 40S consists minimally of a reversible association step during which a thermodynamically stable, yet kinetically dynamic 40S–IRES complex forms, followed by a slow conversion to a kinetically stable [40S–IRES]* complex.

### Mutations in eS25 dramatically affect CrPV IGR IRES activity

**Figure 2.**
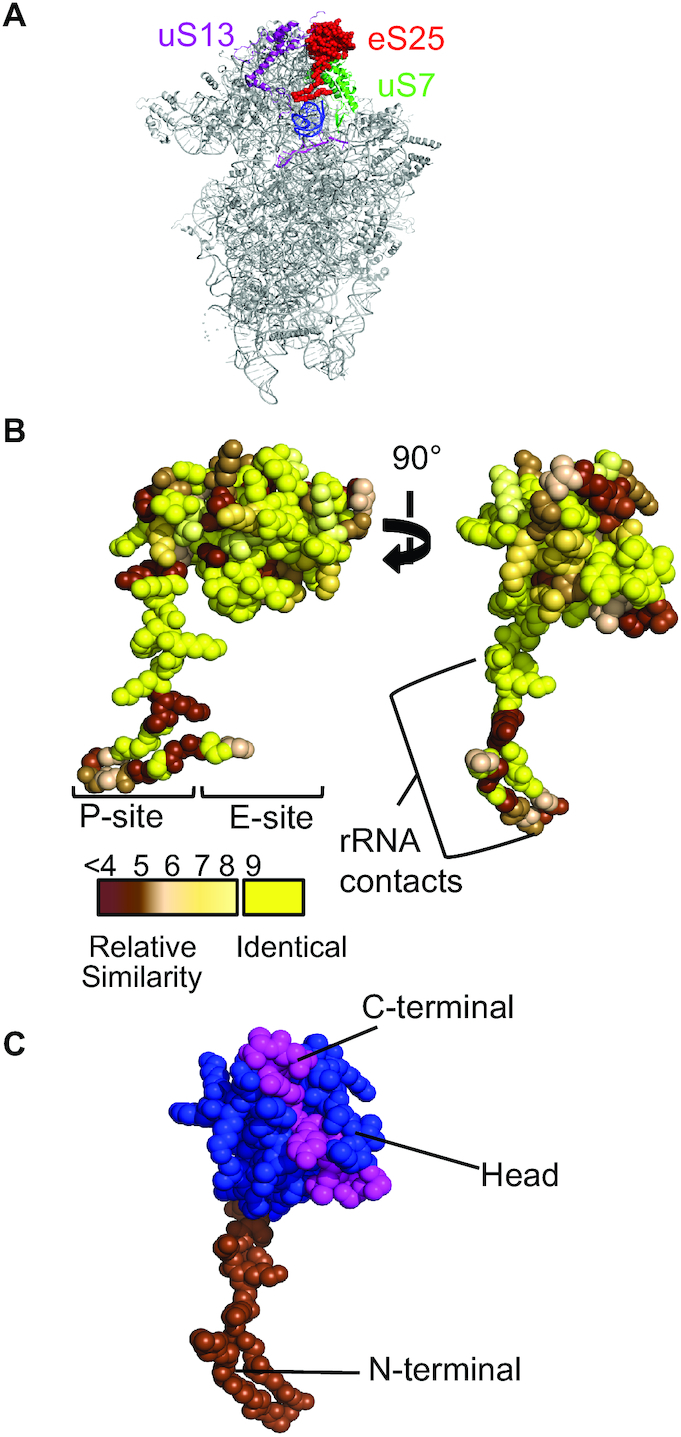
eS25 is a highly conserved non-essential ribosomal protein. (**A**) The globular domain of eS25 (red) is in the E-site of the 40S ribosomal subunit between uS13 (purple) and uS7 (green) with an N-terminal domain extending downward toward the P-site of the 40S ribosomal subunit (blue = tRNA, light purple = mRNA). (**B**) Sequence conservation of eS25 between *S. cerevisiae* and *H. sapiens* modeled onto the *Triticum aestivum* structure of eS25 (4V7E PDB (34)). Identity is indicated using a scale from yellow (identical) to brown (least similar) (35). Regions of eS25 that interact with the P- and E-site, and 40S rRNA are indicated. (**C**) eS25 has three domains: N-terminal (brown; residues 1–29), head (blue; 30–97) and C-terminal (magenta; 98–108) (4V7E PDB (34)).

eS25 is a non-essential 40S ribosomal protein that resides primarily in the E-site with an N-terminal extension that extends towards the P-site (15,23–25) (Figure 2A). Yeast (*S. cerevisiae*) and human (*Homo sapiens*) eS25 coding regions are 49% identical and 71% similar. This conservation extends throughout the entire protein (Figure 2B), which consists of a globular (head) domain, a basic unstructured N-terminal tail domain, and a C-terminal domain that folds into a cleft of the head domain (Figure 2C).

**Figure 3.**
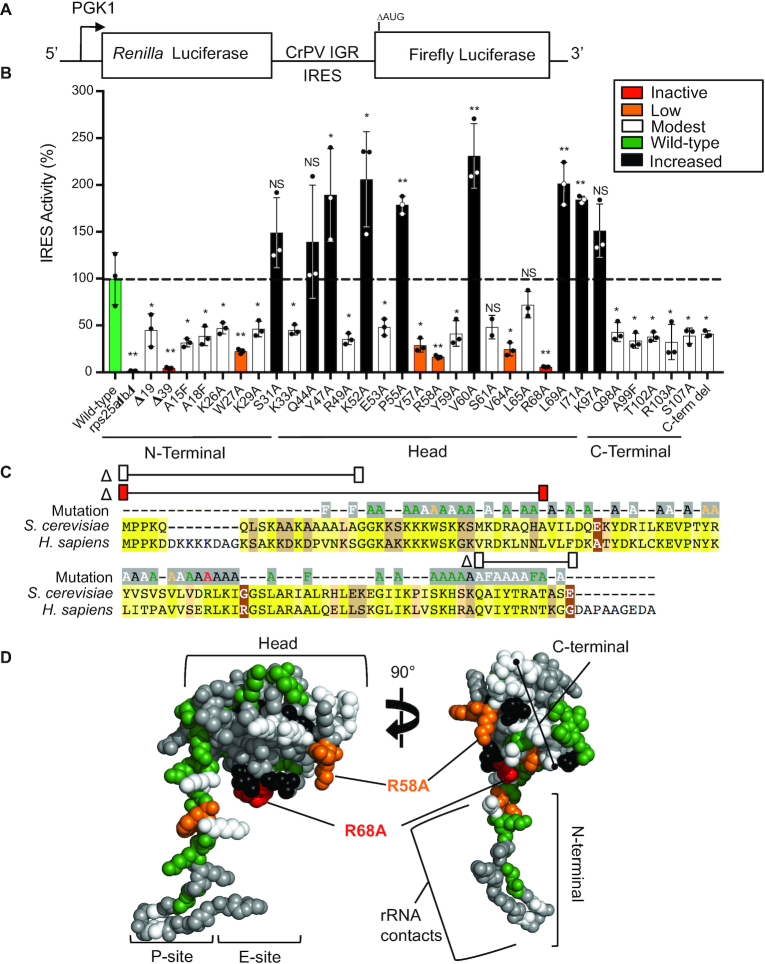
Single point mutations in eS25 dramatically reduced CrPV IGR IRES activity. (**A**) A diagram of the dual luciferase reporter used to assay CrPV IGR IRES activity in yeast. Transcription of the dicistronic reporter is under the control of the *PGK1* promoter; *Renilla* luciferase is translated by a cap-dependent mechanism, and firefly luciferase translation is dependent upon the CrPV IGR IRES, which is ensured by the deletion of the firefly luciferase start codon (ΔAUG). (**B**) A graph of CrPV IGR IRES activity in *rps25ΔaΔb* yeast strain expressing wild-type or mutant eS25 from a plasmid as the sole source of eS25 in the cell. The firefly luciferase values were normalized to *Renilla* luciferase values, and expressed as a percentage of activity with the CrPV IGR IRES in wild-type yeast set to 100%. The color indicates the relative amount of CrPV IGR IRES activity: inactive (red, <10%), low (orange, 17–29%), modest (white, 32–48%), wild-type (green, 72–128%), and increased (black, >139%) activity. Raw luciferase values are shown in Supplementary Table S3. SE is indicated for *n* = 3 biological replicates *P*-value indicated *≤ 0.05, **≤0.01. *P-*values determined by Student's *t*-test. (**C**) Amino acid alignment of *S. cerevisiae* and *H. sapiens* eS25, colored highlights indicate the sequence identity as in Figure 2B. The CrPV IGR IRES activity for each eS25 mutation generated are located above the alignment and the color of the amino acid indicates the CrPV IGR IRES activity for that mutant using the same color code as in (**B**). Amino acid substitutions that showed no significant effect on CrPV IGR IRES activity are shown (green). Three eS25 deletion mutants Δ19 (Δ1–19), Δ39 (Δ1–39), and Δ98–108 are demarcated by a line with colored rectangles above the alignment indicating CrPV IGR IRES activity. (**D**) The CrPV IGR IRES activity of the eS25 mutations mapped onto the structure of eS25 from PDB 4V7E. Colors correspond to their effect on IRES activity as in (*B*), gray indicates residues that were not assayed (see Supplementary Figure S3 for additional views).

Since eS25 is required for CrPV IGR IRES activity and binding to the 40S subunit (15,26), we generated 59 single amino acid mutations in the conserved residues of the yeast eS25 and three deletion mutants (Figure 3C). The mutant eS25 proteins were stably expressed from a plasmid as the sole source of eS25 in the *rps25ΔaΔb* knockout yeast strain and were stably expressed in yeast. Using the dicistronic luciferase assay (Figure 3A) only about half of the mutants affected CrPV IGR IRES activity (Figure 3C and see Supplementary Table S3 for raw luciferase values). Since all of these mutations had IRES activity that was higher than a complete deletion of eS25, this suggests that they were associated with the 40S subunit. In fact, all of the purified 40S subunits had similar levels of eS25 protein associated with the 40S subunits compared to the wild-type (Supplementary Figure S2). Mutations that affected IRES activity were located throughout the entire eS25 protein (Figure 3C, D, and Supplementary Figure S3). Interestingly, deletion of the entire C-terminal domain was no more detrimental to IRES activity than mutating any single amino acid in the C-terminal tail. Deletion of the N-terminal tail or the eS25^R68A or D^ substitutions reduced IRES activity to levels without eS25 (Figure 3B and Supplementary Table S3). Thus, residues throughout the entire protein affected IRES activity, suggesting that eS25 may have multiple roles in CrPV IGR IRES-mediated initiation. Importantly, none of the eS25 mutations affected cap-dependent translation (Supplementary Table S3) consistent with our earlier findings that a deletion of eS25 had no effect on cap-dependent initiation (2,15).

eS25 amino acids R68 and R58 are in close proximity to the CrPV IGR IRES (9,27,28) and are positively charged, suggesting that they may be important for binding to the IRES. Substituting aspartic acid, a negatively charged amino acid, resulted in similar or even lower IRES activities as those seen with the alanine substitution. However, mutation of eS25^R58^ to lysine, another positively charged amino acid, resulted in a partial rescue (Supplementary Table S3) suggesting that a positive charge at that position was important. Mutagenesis of several other residues in this region (Y59 through L65) that are primarily hydrophobic reduced IRES activity (Figure 3B), suggesting that they may stabilize the protein structure such that the positively charged R58 and R68 amino acids are positioned for interacting with the RNA.

### Residues throughout eS25 are required for 40S–IRES complex formation

**Figure 4.**
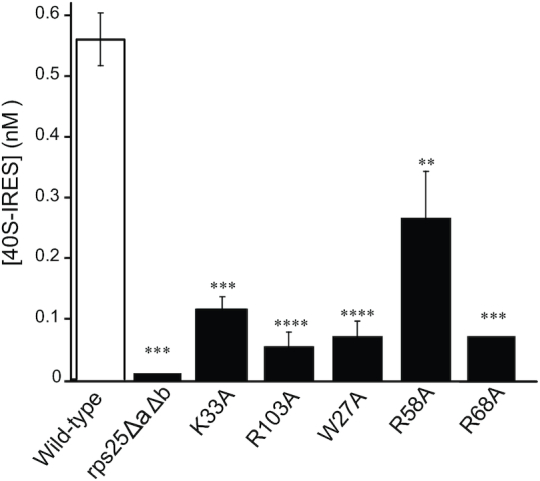
Mutations in eS25 reduce 40S–IRES complex formation. Filter binding assays were performed 3 min after mixing 1 nM of the indicated purified 40S with 4 nM CrPV IGR IRES to determine the amount of 40S–IRES complex formed. 40S–IRES complexes formed using 40S subunits isolated from wild-type (white bar) or *rps25ΔaΔb* are re-presented (Figure 1A) here for direct comparison. Purified 40S subunits from rps25ΔaΔb harboring eS25 expressed from a plasmid as the sole source of eS25 in the cell: K33A, R103A, W27A, R58A and R68A are shown for n≥2 biological repeats with *P*-values indicated *≤0.05, **≤0.01, ***≤0.001 and ****≤0.0001. *P*-values determined by unpaired *t*-test.

Next, we wanted to understand how the eS25 mutations affected the two-step binding reaction to form 40S–IRES complexes. Five mutations in eS25 that were distributed throughout the protein (W27A, K33A, R58A, R68A and R103A) were assayed for effects on IRES binding by purifying 40S subunits from yeast harboring the mutant eS25 as the only source of eS25 in the cell. All 40S subunits with mutant eS25 had decreased binding of the IRES to the 40S (Figure 4). However, IRES binding was clearly detectable in each case and is thus markedly higher with mutant eS25 than without eS25 (Figure 4). These results show that eS25 mutations diminish 40S–IRES complex formation *in vitro*.

### eS25 mutations affect both steps of the 40S–IRES binding reaction

**Figure 5.**
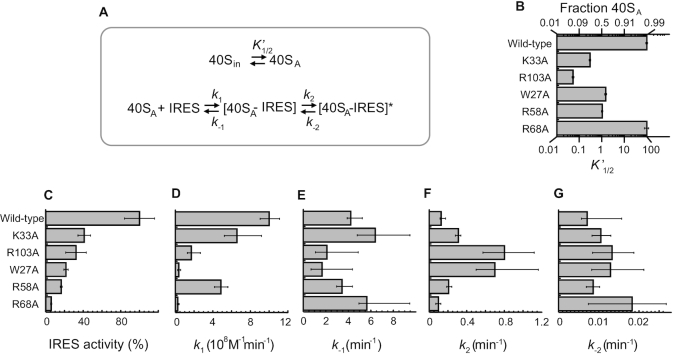
Impact of eS25 mutations on rate and equilibrium constants of the 40S–IRES formation. *(***A***)* Minimal kinetic model for the two-step binding reaction to form a 40S-CrPV IGR IRES complex. For the top part of the model the terms are defined as follows: (40S_in_): the inactive 40S-eS25 conformation that cannot bind IRES RNA, (40S_A_): the active 40S-eS25 that can readily bind IRES RNA, *K*’_1/2_: equilibrium constant for the 40S_in_ to 40S_A_ transition, defined as: (fraction of 40S_A_) / (1- fraction of 40S_A_). The bottom part of the model and the corresponding terms are the same as defined in Figure 1F. (B–G) Graphs showing parameters for wild-type and mutant 40S_A_-eS25 as follows: (**B**) equilibrium constant *K*’ $\frac{1}{2}$ (for 40S_in_ to 40S_A_ transition) along with fraction of active 40S, (**C**) IRES activity (%) from Figure 1F presented again here for clarity, (**D**) association rate constants (k_1_) for the binding of 40S_A_ to the IRES, (**E**) Dissociation rate constants (k_-1_) for the dissociation of the 40S_A_-IRES complex, (**F**) forward conformational change rate constant (*k*_2_) for the transition from [40S_A_-IRES] to [40S_A_-IRES]*, (**G**) Reverse conformational change rate constant (*k*_–2_) for the transition from [40S_A_-IRES]* to [40SA-IRES]. For plots (B–G) error bars represent the upper and lower boundaries for each of the kinetic parameters at the 95% confidence interval as determined by *FitSpace* analysis.

The association and dissociation rates of the 40S–IRES complex with each mutant eS25 displayed a marked biphasic shape under all conditions (Supplementary Figure S4), consistent with a two-step reversible reaction. However, deviations in the curves from wild-type eS25 suggested that the various eS25 mutations had different effects on each step in the reaction. Importantly, the IRES–40S complexes formed with the eS25 mutants were competent to form stable 80S complexes (Supplementary Figure S5). We determined the rate constants for each of the eS25 mutations using the two-step binding reaction established for wild-type eS25. In order to accomplish an adequate data fit for all eS25 mutants, we included an additional step that accounts for a slow equilibrium between a form of the 40S that is competent for IRES binding (40S_A_; active) and one that is not (40S_in_; inactive) (Figure 5A). This augmented kinetic scheme was used to globally fit association and dissociation time courses (Supplementary Figure S4).

The eS25^R68A^ mutation, which caused the largest decrease in IRES activity *in vivo*, reduced the binding rate constant for the first step (*k*_1_) by a factor of 40 (Figure 5C and D), and increased the back-reaction rate constant for the second step (*k*_–2_) by a factor of ∼2, compared to wild-type eS25 (Figure 5G, and Supplementary Table S2). The other two rate constants were roughly similar to wild-type eS25. eS25^R68A^ also did not significantly alter the equilibrium between 40S that were competent for IRES binding and 40S that were not, compared to wild-type eS25 (Figure 5B). These findings suggest that while eS25^R68A^ reduced the affinity of the IRES for the 40S subunit, it did not alter the conformation of the 40S subunit to one that was not conducive to binding.

In contrast to eS25^R68A^, all other eS25 mutations shifted the equilibrium towards non-competent IRES binding 40S (Figure 5B). All mutations reduced the binding rate constant for the first step (*k*_1_), albeit to varying degrees (Figure 5D). No comparably clear trends were seen for the impact of the mutations on *k*_–1_ (Figure 5E). However, except for eS25^R68A^, the mutations increased the rate constant for the second step (*k*_2_) (Figure 5F), and all mutations increased the reverse rate constant *k*_–2_ (Figure 5G). Collectively, these data show that mutations affect all steps of the binding reaction and also impact the fraction of 40S that is competent for IRES binding.

A decrease in *k*_1_ and an increase in *k*_-2_ and *k*_2_ appear to be detrimental to IRES binding *in vitro*. These observations indicate that diminished association rate constant and altered timing of formation of the second, stable [40S_A_-IRES]* complex have the main kinetic impact on 40S–IRES complex formation. Mutations that accelerate [40S_A_-IRES]* formation are detrimental, as are mutations that promote the conversion of this complex back to the intermediate [40S_A_-IRES] complex. With the exception of the eS25^R58A^ mutation, the measured kinetic effects on *k*_1_ and *k*_2_ scale with the impact of the mutations on the IRES activity measured *in vivo* (Figure 5C, D and F). This observation suggests that the slower timing of [40S_A_-IRES]* formation is important for IRES activity.

### eS25 impacts 40S conformation in the absence of the IRES

**Figure 6.**
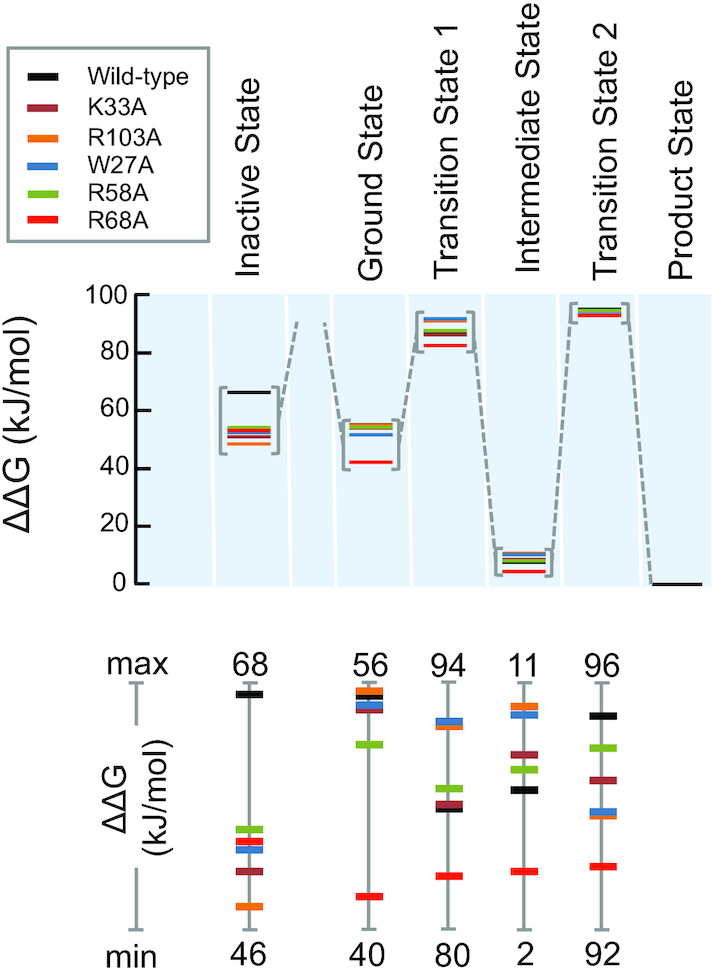
Impact of eS25 mutations on the free energy landscape of the 40S–IRES binding reaction. Relative free energies (ΔΔ*G*) for the reaction stages of the 40S–IRES binding reaction for wild-type and mutant eS25, as marked. The product state was set equal for all eS25 variants. The lower panel shows free energy changes for the various stages at a larger scale, emphasizing differences between the eS25 variants. Note the different scales.

To better visualize the impact of eS25 on the timing of [40S_A_-IRES]* formation, we converted the rate constants into a free energy diagram for each eS25 variant. Assuming that the [40S_A_-IRES]* complex is critical for IRES activity we anchored the free energies for each eS25 variant at this state (Figure 6). The diagram reveals that all tested mutations stabilize the 40S state that is not competent to bind the IRES (Inactive state; 40S_in_) with eS25^R103A^ stabilizing the inactive state the most. These observations reiterate that all tested eS25 mutated residues impact the 40S by favoring a conformation that is not conducive to IRES binding, which persists on the timescale of our experiments.

The ground state energy of the 40S that is competent for IRES binding (40S_A_) is significantly lower for eS25^R68A^ compared to wild-type and changes only little for the other mutations. This ground state energy also reflects the impact of eS25 on the 40S conformation. A lower energy for the ground state disfavors IRES binding, because the association rate constant for the first step is determined by the difference between ground and transition state. With the exception of eS25^R68A^ all mutations increased or slightly increased the transition state energy, compared to wild-type. Thus, eS25^R68A^ favors the transition state while all other mutations destabilized it, to varying degrees. However, the favorable impact of the eS25^R68A^ mutation was outweighed by the unfavorable impact of this mutation on the ground state. Nevertheless, the effect of the mutations on ground and transition state energies for the first step marked the major energetic impact on the overall reaction. The intermediate state [40S_A_-IRES] was destabilized by all mutations, compared to wild-type, except for eS25^R68A^, which stabilized this state. The transition state for the second step is highest for the wild-type. These findings suggest that, in contrast to step 1, step 2 does not need to be as fast as possible. A slow conversion of the intermediate state [40S_A_-IRES] to the final product state [40S_A_-IRES]* seems to be most desirable for optimal IRES activity.

## DISCUSSION

**Figure 7.**
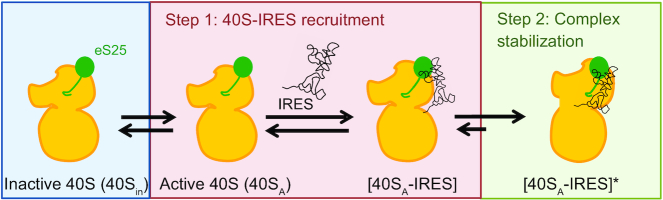
Model of the two-step reaction of IRES recruitment of the 40S subunit and the role of eS25 in this process. Wild-type eS25 is required for inducing a conformation in the 40S subunit that is conducive for IRES binding (blue). The first step is a fast-bimolecular binding reaction where the IRES binds to the 40S subunit but does not form a stable interaction. This complex can be readily dissociated (red). The second step is a slow unimolecular reaction to form a distinct complex that is more stable, likely this represents a conformational change in the 40S–IRES complex possibly tilting the head away from the body of the 40S subunit, which would open the mRNA binding channel for positioning of the IRES PKI into the A-site. eS25 is important for slowing down this step or preventing it from occurring prematurely. This final step may represent the 40S–IRES complex that has been observed in structural studies with the CrPV IGR IRES loaded into the mRNA channel (6,7,13).

Taken together, these studies revealed that CrPV IGR IRES binding is a reversible two-step reaction that can be described as IRES binding to the 40S subunit followed by a conformational change that stabilizes the complex (Figure 7). The presence of wild-type eS25 on the 40S subunit induces a conformation that is suitable for IRES binding. In addition, the R68 residue of eS25 was critical for CrPV IGR IRES binding to the 40S subunit and mutations that reduced the rate of IRES association with the 40S subunit correlated with a decrease in IRES activity *in vivo*, suggesting that this is the rate-limiting step for IRES activity. Our studies revealed multiple roles for eS25 in CrPV IGR IRES-mediated initiation and identified residues in eS25 that affect these roles. eS25 is important for inducing a binding competent 40S subunit and ensuring that the conformational change that stabilizes binding does not occur too rapidly or prematurely.

Although the CrPV IGR IRES has been reported to contact eS25, eS28, uS7 and uS11 (8), eS25 is critical for this initial binding step. Our data show that the integrity of R68 in eS25 is particularly critical for efficient IRES binding to the 40S, both *in vitro* and in the cell. This finding is consistent with the location of eS25 on the 40S subunit and structural studies indicating that SL2.3 (SLV) of the CrPV IGR IRES contacts eS25 (9,27) (Supplementary Figure S6). R68 is a positively charged residue that could facilitate interactions with a negatively charged RNA since it is on a surface exposed region of eS25. The large energetic impact of the R68A mutation is consistent with the possibility that R68 directly contacts the IRES.

The data presented here showed that CrPV IGR IRES binding to the 40S is a two-step reaction. Previous studies that only detected a single step determined 40S–IRES complex formation to be very slow (29). This is consistent with the second step measured in our experiments and suggest that previous measurements, which were based on a FRET system, were not sensitive to the first and faster reaction step. This insensitivity could arise from an unsuitable distance between the FRET labels until after the initial binding step or if the initial binding state consists of a multitude of dynamically changing IRES orientations, which would not produce a uniform FRET state. Also, a combination of both scenarios could explain the discrepancies between our studies and those measured by FRET. However, in agreement with our findings, the FRET study showed that the reversal of the slow step did not occur in the time scale of initiation (29), suggesting that the stable complex [40S_A_-IRES]* does not dissociate prior to initiation.

We speculated that the second step likely represents a conformational change of the 40S–IRES complex. An empty 40S subunit has a ‘closed’ mRNA channel with the head positioned close to the body (24). Rotation of the head away from the body accompanied by the presence of initiation factors (eIF1, eIF1A, eIF3 and the ternary complex) results in opening the latch for mRNA loading and/or scanning, which is referred to as the ‘open’ conformation (30). Structural studies have shown that binding of the CrPV IGR IRES to 40S subunit with PKI positioned into the A-site (13,27) in the absence of any other factors induces the open conformation with the head rotated away from the body of the 40S for mRNA loading (6,7). Therefore, it is possible that the conformational change reflected in the second reaction step involves the head movement that opens the mRNA channel, which would allow binding of PKI of the CrPV IGR IRES into the A-site of the 40S subunit (Figure 7). It is tempting to speculate that IRES binding to the 40S subunit only becomes stable once the IRES is positioned into the mRNA channel. However, at this point we are unable to rule out a multi-step reaction whereby opening of the mRNA channel is followed by recognition of PKI in the A-site as a mRNA-tRNA mimic (27), which has been shown to induce a ‘domain closure’ whereby the beak of the 40S subunit moves toward the body by 4 Å (27,31).

The rate for the reversal of the second step is slow (*k*_–2_ ∼ 0.01 min^−1^) on the time scale of initiation, which is thought to take about 60 s, making it essentially irreversible (32). However, initiation from the IRES is much less efficient and likely much slower than canonical initiation and the rate of initiation of the CrPV IGR IRES might be more accurately measured by the rate of the first pseudotranslocation event, which occurs with a rate constant of *k*_init_ ∼ 0.2 min^−1^ (33). Yet, even in this scenario, initiation is faster than the reversal of the second step (*k*_–2_). Therefore, the quantitative biochemical data suggest that once the IRES recruits the 40S subunit and induces the putative conformational change, the complex is unlikely to dissociate before initiation is completed and elongation commences.

The correlation between the rate constant for the first step (*k*_1_) and the IRES activity holds for all tested mutations, except for eS25^R58A^ (Figure 5). Previous studies have implicated R58 along with R68 in IRES binding since R58 resides on the same surface exposed region of eS25 (28). However, our kinetic analysis indicates only comparably minor effects of eS25^R58^ on both steps of the binding process (Figure 6). We therefore speculate that the large effect of eS25^R58^ on IRES activity in cells arises because eS25^R58^ is likely required for a step downstream of the IRES binding process, such as one or more translocation steps. Indeed, the double translocated IRES in the 80S ribosome has been shown to make new contacts with eS25 residues 52–65 of the mammalian eS25 (residues 50–57 yeast eS25) (9), suggesting that the alpha-helix containing R58 is important downstream of 40S complex formation.

## Supplementary Material

gkaa547_Supplemental_File

## Notes

Present address: Beth Walters, Microbiology, New York University, New York, NY 10016, USA.
