# Supplementary material for: Binding of a viral IRES to the 40S subunit occurs in two successive steps mediated by eS25
Source: Nucleic Acids Res. 2020 Jul 1;48(14):8063–73. doi: 10.1093/nar/gkaa547 (PMC7430650; doi:10.1093/nar/gkaa547)

Supplementary tables and figures

Supplementary Table 1. Primer list for generating eS25 mutants.

Supplementary Figure 1. Time courses are best described by a kinetic model consisting of two consecutive reactions. (*A*) The [40S-IRES] complex is formed in a single step. (*B*) 40S and IRES form two separate 40S-IRES complexes in two distinct parallel reactions. (*C*) Two populations of 40S bind IRES form two separate 40S-IRES complexes in two distinct reactions. (*D*) The final [$40S_A$-IRES]* complex forms by two consecutive reactions, initial 40S and IRES binding [$40S_A$-IRES] and conversion to the final complex. (*E*) Goodness of fit parameters for global fitting of the time courses for wild-type and mutant eS25 to each of the four models described above. [σ fit]: standard deviation of the data, a smaller value indicates a better fit. [X^2]: total chi^2 for global fit (normalized to average sigma value), a smaller value indicates a better fit. [X^2 / DoF, DoF: degrees of freedom]: measure of goodness of fit, a value of 1 indicates a perfect fit. The smaller the value, the better the fit. [σ fit]: standard deviation of the data, a smaller value indicates a better fit. Model (*D*) yields the best fit parameters and was therefore considered for data analysis.

Supplementary Table 2. Upper and lower boundaries for each of the four kinetic parameters at the 95% confidence interval. Parameters are defined as in the text; k_1: association rate constant; k_{-1}: dissociation rate constant; k_2: forward rate constant for step 2; k_{-2}: reverse rate constant for step 2.

Supplementary Table 3. The CrPV IGR IRES (firefly luciferase) and cap-dependent (*Renilla* Luciferase) activity measured using the in *rps25ΔaΔb* yeast strain expressing the wild-type or mutant eS25 from a plasmid as the sole source of eS25 in the cell. SD for n=3 biological repeats are indicated. Also, shown are the relative levels of eS25 in whole cell lysates from yeast strains expressing eS25 mutants which were determined by quantitative western analysis of eS25

levels normalized to PGK levels and expressed relative to wild-type which was set to 100%. p-values were determined by a Student's *t*-test.

Supplementary Figure 2. Purified yeast ribosomal subunits contain eS25. Western analysis of ribosomal subunits isolated from yeast strains expressing wild-type eS25, *rps25ΔaΔb, or rps25ΔaΔb* yeast expressing the indicated mutant eS25 from a plasmid. The eS25 levels were quantified relative to the control, ribosomal protein S6 (eS6) and normalized to wild-type. Shown is representative of n = 3.

Supplementary Figure 3. The CrPV IGR IRES activity of the eS25 mutations mapped onto the structure of eS25 from PDB 4V7E. Colors represent CrPV IGR IRES activity: dead (red, <10%), low (orange, 17-29%), modest (white, 32-48%), wild-type (green, 72-128%), and increased (black, >139%) activity, and gray indicates residues that were not assayed.

Supplementary Figure 4. Association and dissociation of 40S-IRES complexes with mutant eS25 (*A*) Time-courses for 40S-IRES association. The concentration of [40S-eS25] is indicated in the panels. Concentration of [IRES] = 1 nM (●), 2.5 nM (○) and 4 nM (♦); except for R103A, where [IRES] = 2 nM (●),5 nM (○) and 8 nM (♦). 40S and IRES concentrations were chosen to accomplish similar experimental time frames for the different eS25 mutations. Lines show the global fit to the two-step kinetic model described in Fig 1F, augmented with an equilibrium step for interconversion between 40S subunits that are competent for IRES binding and 40S subunits that are not (Fig 5A). **(*B*)** Time-courses for IRES dissociation from the 40S subunit (([40S-eS25] = 2 nM, [IRES] = 2 nM, except for R68A, where (([40S-eS25] = 8 nM, [IRES] = 8 nM. [Chase RNA] = 900 nM for all experiments). Error bars indicate one standard deviation of multiple (n ≥ 3)

independent experiments. Lines show the global fit to the kinetic model described in Fig 5A**.**

Supplementary Figure S5. 60S subunit joining stabilizes 80S complex formation. ^{32}P-labelled wild-type (solid lines) or mutant (cc-gg; dotted lines) CrPV IGR IRES RNA was incubated with purified 40S subunits with the indicated eS25 mutations ((A-F): Wild-type, W27A, K33A, R58A, R68A, R103A, respectively). Then 60S subunits were added (gray) or not (black) and the 80S-IRES complexes were allowed to form for 20 minutes before 450-fold excess unlabeled wild-type CrPV IGR IRES was added to compete off labeled CrPV IGR IRES. Maximal complex formation before competitor was added (0 minutes) was set to 1, complexes were measured by filter binding assays from 1 to 60 minutes post competitor RNA addition.

Supplementary Figure 6. eS25 residues create binding pocket for CrPV IGR IRES. The binding surface of the CrPV IGR IRES (blue) to the 40S subunit (gray) in relation to eS25 (red) from PDB 5IT9. eS25 residues (Y57, R58, V64, and R68) form a pocket-like structure for SL2.3 of the CrPV IGR IRES to insert into.

Supplementary Table 1. Primer list for generating eS25 mutants.

	Primer (5'-3')	
**Mutant** *rps25a*	Forward	Reverse
A15F	AAAGCCGCTTTTGCCCTTGC	GCAAGGGCAAAAGCGGCTTT
A18F	CTGCTGCCCTTTTCGGTGGTAAGAA	TTCTTACCACCGAAAAGGGCAGCAG
G20A	GCTGCCCTTGCTGGTGCTAAGAAGTC	CTTCTTAGACTTAGCACCAGCAA
K21A	CTGCTGCCCTTGCTGCTGGTGCTAAG	GACCACTTCTTCTTAGACTTAGCACC
K24A	GCTGGTGGTAAGAAGTCTGCTAAGAA	CTTTTTGGACCACTTCTTAGCAGACTT
K25A	GGTGGTAAGAAGTCTAAGGCTAAGTG	GGACTTTTTGGACCACTTAGCCTTAG
K26A	GTGGTAAGAAGTCTAAGAAGGCGTG	CATGGACTTTTTGGACCACGCCTTCT
W27A	GGTAAGAAGTCTAAGAAGAAGGCTTC	GTCTTTCATGGACTTTTTGGAAGCCTT
W27F	GTGGTAAGAAGTCATAGAAGAAGTTT	TCTGTCTTTCATGGACTTTTTGGAAAA
W27L	CTAAGAAGAAGCTCTCCAAAAAGTC	GACTTTTTGGAGAGCTTCTTCTTAG
S28A	GTAAGAAGTCTAAGAAGAAGTGGGCT	CTGTCTTTCATGGACTTTTTAGCCCAC
K29A	GAGCTCTGTCTTTCATGGACTTAGCG	GAAGTCTAAGAAGAAGTGGTCCGCTA
S31A	GAAGAAGTGGTCCAAAAAGGCTATGA	GTGTTGAGCTCTGTCTTTCATAGCCT
K33A	GAAGTGGTCCAAAAAGTCCATGGCTG	GACAGCGTGTTGAGCTCTGTCAGCCA
R35A	CATGAAAGACGCAGCTCAACAC	GTGTTGAGCTGCGTCTTTCATG
Q37A	AAGACAGAGCTGCTCACGCTGTCAT	ATGACAGCGTGAGCAGCTCTGTCTT
H38A	ACAGAGCTCAAGCAGCTGTCATTTT	AAAATGACAGCTGCTTGAGCTCTGT
V40A	GACAGAGCTCAACACGCTGCTATTTT	ACTTCTCTTGGTCTAAAATAGCAGCG
Q44A	TCATTTTAGACGCAGAGAAGTACG	CGTACTTCTCTGCGTCTAAAATGA
Y47A	ACCAAGAGAAGGCAGACAGAATCTT	AAGATTCTGTCTGCCTTCTCTTGGT
R49A	AGAAGTACGACGCCATCTTGAAGGA	TCTTCATGCTGCGGTAGAACTTCCT
K52A	ACAGAATCTTGGCAGAAGTTCCAAC	GTTGGAACTTCTGCCAAGATTCTGT
E53A	CGACAGAATCTTGAAGGCTGTTCCAA	CGTATCTGTAAGTTGGAACAGCCTTC
P55A	GACAGAATCTTGAAGGAAGTTGCTAC	GACAGAAACGTATCTGTAAGTAGCAA
Y57A	GAATCTTGAAGGAAGTTCCAACTGCT	CAAAACAGAGACAGAAACGTATCTAG
R58A	GACCAAAACAGAGACAGAAACGTATG	GAATCTTGAAGGAAGTTCCAACTTAC
R58D	TTCCAACTTACGACTACGTTTCTGT	ACAGAAACGTAGTCGTAAGTTGGAA
R58K	CTTGAAGGAAGTTCCAACTTACAAAT	CAAAACAGAGACAGAAACGTATTTGT
R58Q	TTCTGTCTCTGTTTTGGTCGACCAATT	CTAAAGAACCACCAATCTTTAATTGGT
Y59A	CAACTTACAGAGCTGTTTCTGTCTC	GAGACAGAAACAGCTCTGTAAGTTG
V60A	TTACAGATACGCGTCTGTCTCTGT	ACAGAGACAGACGCGTATCTGTAA
S61A	CAGATACGTTGCTGTCTCTG	CAGAGACAGCAACGTATCTG
V62A	ATACGTTTCTGCGTCTGTTTTGGT	ACCAAAACAGACGCAGAAACGTAT

V64A	TCTGTCTCTGCATTGGTCGACA	TGTCGACCAATGCAGAGACAGA
L65A	TGTCTCTGTTGCGGTCGACAGA	TCTGTCGACCGCAACAGAGACA
V66A	CTCTGTTTTGGCCGACAGATTA	TAATCTGTCGGCCAAAACAGAG
D67A	CTGTTTTGGTCGCGAGATTAAAGAT	ATCTTTAATCTCGCGACCAAAACAG
R68A	GTTTTGGTCGACGCATTAAAGATTG	CAATCTTTAATGCGTCGACCAAAAC
R68D	TTTTGGTCGACGACTTAAAGATTGG	CCAATCTTTAAGTCGTCGACCAAAA
L69A	GTCTCTGTTTTGGTCGACAGAGCAAA	GCTAAAGAACCACCAATCTTTGCTCT
K70A	CTGTTTTGGTCGACAGATTAGCGATT	CTAGCTAAAGAACCACCAATCGCTAA
I71A	ACAGATTAAAGGCTGGTGGTTCTT	AAGAACCACCAGCCTTTAATCTGT
L75A	GATTAAAGATTGGTGGTTCTGCTGCT	GTGTCTCAAAGCAATTCTAGCAGCAG
G87A	GGAAAAGGAAGCAATCATCAAGCC	GGCTTGATGATTGCTTCCTTTTCC
I89A	AAAAGGAAGGTGCGATCAAGCCAAT	ATTGGCTTGATCGCACCTTCCTTTT
S93A	TCAAGCCAATCGCAAAGCACTCCAA	TTGGAGTGCTTTGCGATTGGCTTGA
K94A	GGTATCATCAAGCCAATCTCCGCTCA	GTAGATATCTTGCTTGGAGTGAGCGG
H95A	ATCATCAAGCCAATCTCCAAGGCTTC	GGTGTAGATAGCTTGCTTGGAAGCCT
S96A	CAAGCCAATCTCCAAGCACGCTAAGCAAGCTATCT ACACCAG	CTGGTGTAGATAGCTTGCTTAGCGTGCTTGGAGATT GGCTTG
K97A	CCAATCTCCAAGCACTCCGCTCAAGC	CTCTGGTGTAGATAGCTTGAGCGGAG
Q98A	AGCACTCCAAGGCTGCTATCTACA	GTGTAGATAGCAGCCTTGGAGTGCT
A99F	ACTCCAAGCAATTCATCTACACCAG	CTGGTGTAGATGAATTGCTTGGAGT
I100A	CCAAGCAAGCTGCATACACCAGAGC	GCTCTGGTGTATGCAGCTTGCTTGG
Y101A	CAAGCTATCGCCACCAGAGCT	AGCTCTGGTGGCGATAGCTTG
T102A	AGCTATCTACGCCAGAGCTA	TAGCTCTGGCGTAGATAGCT
R103A	TATCTACACCGCAGCTACTGCT	AGCAGTAGCTGCGGTGTAGATA
A104F	CTACACCAGATTTACTGCTTCT	AGAAGCAGTAAATCTGGTGTAG
T105A	ACCAGAGCTGCTGCTTCTG	CAGAAGCAGCAGCTCTGGT
S107A	AGAGCTACTGCTGCTGAA	TTCAGCAGCAGTAGCTC
ΔC-terminal	AAGCACTCCAAGTAAGCTATCTAC	TGTAGATAGCTTACTTGGAGTGCTT

Supplementary Table 2 Upper and lower boundaries for each of the four kinetic parameters at the 95% confidence interval

40S Identity	k_1 ($M^{-1}min^{-1}$)	Lower bound	Upper bound	k_{-1} (min^{-1})	Lower bound	Upper bound	k_2 (min^{-1})	Lower bound	Upper bound	k_{-2} (min^{-1})	Lower bound	Upper bound
Wild-type	$1.00 \cdot 10^9$	$9.05 \cdot 10^8$	$1.11 \cdot 10^9$	4.21	3.90	5.23	0.127	0.115	0.175	0.0071	0.0057	0.0157
K33A	$6.57 \cdot 10^8$	$5.23 \cdot 10^8$	$9.19 \cdot 10^8$	6.39	4.79	9.48	0.311	0.279	0.338	0.0105	0.0083	0.0130
R103A	$1.70 \cdot 10^8$	$1.29 \cdot 10^8$	$2.66 \cdot 10^8$	2.09	1.08	4.88	0.803	0.573	1.12	0.0133	0.0085	0.0187
W27A	$3.69 \cdot 10^7$	$2.52 \cdot 10^7$	$5.66 \cdot 10^7$	1.66	0.67	4.37	0.699	0.501	1.16	0.0129	0.0083	0.0212
R58A	$4.85 \cdot 10^8$	$4.19 \cdot 10^8$	$5.60 \cdot 10^8$	3.43	2.96	4.37	0.207	0.187	0.241	0.0086	0.0068	0.0102
R68A	$2.50 \cdot 10^7$	$2.22 \cdot 10^7$	$3.94 \cdot 10^7$	5.64	4.95	9.46	0.097	0.071	0.124	0.0183	0.0075	0.0268

Supplementary Table 3. eS25 expression levels and dual-luciferase raw values in yeast

Mutant eS25	Percent IRES[a] Activity	Percent eS25 Protein[b] (%)	Firefly Luciferase Raw Values (RLUs)[c] ($x10^3$)	*Renilla* Luciferase Raw Values (RLUs) ($x10^6$)[c]	*p-value*
Wild-type	100	100	33±6	7.6±1.2	-
rps25ΔaΔb	2	0	0.64±0.053	7.6±2.0	0.01
Δ19	45	233	15±4.0	5.0±2.0	0.02
Δ39	5	63	0.63±0.019	3.0±0.5	0.01
A15F	32	173	11±2.0	10.0±0.2	0.02
A18F	39	179	11±0.4	9.1±1.1	0.04
G20A	126	89	33±2.0	7.9±0.49	0.23
K21A	72	100	23±6.0	9.3±1.2	0.09
K24A	74	111	8.0±1.0	8.4±0.44	0.11
K25A	95	128	9.0±0.4	6.6±0.08	0.41
K26A	47	100	4.5±0.6	5.5±0.63	0.04
W27A	22	115	2.0±0.2	5.4±0.63	0.02
W27F	34	62	13±1.5	11.0±1.4	0.04
W27L	20	55	6±0.2	5.9±0.39	0.02
S28A	110	177	20±2.0	5.5±0.23	0.28
K29A	46	145	11±1.0	7.0±0.61	0.06
S31A	123	21	33±9	4.5±0.97	0.31
K33A	41	145	13±1.0	6.6±0.70	0.06
R35A	101	98	16±0.7	4.2±0.55	0.46
Q37A	114	152	31±3.0	8.6±1.4	0.07

H38A	114	54	28±2.0	7.6±0.25	0.23
V40A	299	165	33±0.5	3.8±1.0	0.08
Q44A	139	130	40±3.0	6.5±1.3	0.26
Y47A	189	112	44±4.0	4.9±0.35	0.08
R49A	36	189	12±0.7	11.0±0.55	0.02
K52A	206	134	32±1.0	3.3±0.70	0.05
E53A	48	105	29±2.0	6.4±1.7	0.06
P55A	178	81	26±1.8	10±1.3	0.02
Y57A	29	130	6±0.2	4.8±0.75	0.03
R58A	29	89	6.0±0.8	9.0±0.10	0.02
R58D	5	97	2.1±0.1	9.9±0.46	0.01
R58K	60	129	19±0.5	9.4±1.1	0.05
R58Q	34	129	13±1.0	8.7±0.73	0.02
Y59A	41	61	16±1.5	8.3±0.7	0.01
V60A	231	78	51±0.13	5.0±0.4	0.03
S61A	48	76	29±2	6.4±1.6	0.06
V62A	96	75	21±0.9	6.9±1.1	0.44
V64A	25	121	7.5±2.0	6.7 ±0.71	0.03
L65A	40	98	27±2.0	7.8±1.6	0.05
V66A	103	54	45±3.0	9.1±0.31	0.38
D67A	204	72	80±8.6	9.1±1.3	0.02
R68A	6	96	2.0±0.42	6.0±0.68	0.01
R68D	2	67	1.0±0.04	9.6±0.46	0.01
L69A	201	157	21±2.3	6.1±0.31	0.003
K70A	72	100	13±2.0	6.0±0.74	0.09
I71A	184	80	47±3.0	7.5±0.41	0.01
L75A	72	126	20±0.84	8.1±0.54	0.18
G87A	120	192	33±0.5	8.4±0.53	0.16
I89A	78	100	35±0.7	9.6±1.3	0.24

S93A	92	211	41±3.0	9.1±0.43	0.28
K94A	84	105	26±1.0	9.2±0.33	0.20
H95A	97	181	40±3.0	8.5±2.0	0.46
S96A	113	152	33±5.0	8.6 ±0.65	0.25
K97A	151	70	42±0.9	8.4±0.86	0.01
Q98A	43	64	31±1.0	7.2±0.15	0.04
A99F	34	46	25±3.0	7.6±0.44	0.04
I100A	59	132	42±0.7	9.0±0.30	0.07
Y101A	63	165	30±1.0	10±1.2	0.02
T102A	38	130	11±0.4	6.1±0.54	0.03
R103A	32	110	10±2.0	7.0±0.95	0.06
A104F	72	111	50±4.0	7.8±1.6	0.05
T105A	88	172	31±2.0	7.2±0.52	0.26
S107A	39	90	23±2.0	6.0±0.58	0.02
ΔC-terminal	41	96	18±0.2	9.0±0.30	0.03

[a] Percent of CrPV IGR IRES activity relative to CrPV IGR IRES activity of wild-type eS25 set to 100%.
[b] Percent of mutant eS25 protein relative to wild-type eS25 protein levels set to 100%.
[c] SD for n=3 is shown.

A

$$40S_A + IRES \underset{k_{-1}}{\overset{k_1}{\rightleftharpoons}} [40S_A\text{-}IRES]$$

B

$$40S_A + IRES \underset{k_{-1}}{\overset{k_1}{\rightleftharpoons}} [40S_A\text{-}IRES]$$

$$40S_A + IRES \underset{k_{-2}}{\overset{k_2}{\rightleftharpoons}} [40S_A\text{-}IRES]^*$$

C

$$40S_A + IRES \underset{k_{-1}}{\overset{k_1}{\rightleftharpoons}} [40S_A\text{-}IRES]$$

$$40S^*_A + IRES \underset{k_{-2}}{\overset{k_2}{\rightleftharpoons}} [40S_A\text{-}IRES]^*$$

D

$$40S_A + IRES \underset{k_{-1}}{\overset{k_1}{\rightleftharpoons}} [40S_A\text{-}IRES] \underset{k_{-2}}{\overset{k_2}{\rightleftharpoons}} [40S_A\text{-}IRES]^*$$

E

	X^2				X^2 / DoF				σ fit			
**Model**	**WT**	**R58A**	**R68A**	**W27A**	**WT**	**R58A**	**R68A**	**W27A**	**WT**	**R58A**	**R68A**	**W27A**
**(a)**	7839	157	282	86.8	218	3.91	4.41	2.06	0.502	0.0313	0.111	0.0917
**(b)**	140	61.2	380	83.7	4.12	1.61	6.23	2.09	0.0563	0.0236	0.0447	0.0610
**(c)**	111	166	385	92.8	3.48	4.61	6.42	2.44	0.0565	0.0699	0.171	0.0917
**(d)**	131	59.2	98.0	54.5	3.43	1.51	1.66	1.47	0.0509	0.0216	0.0229	0.0341

Walters_Supplementary Fig S2

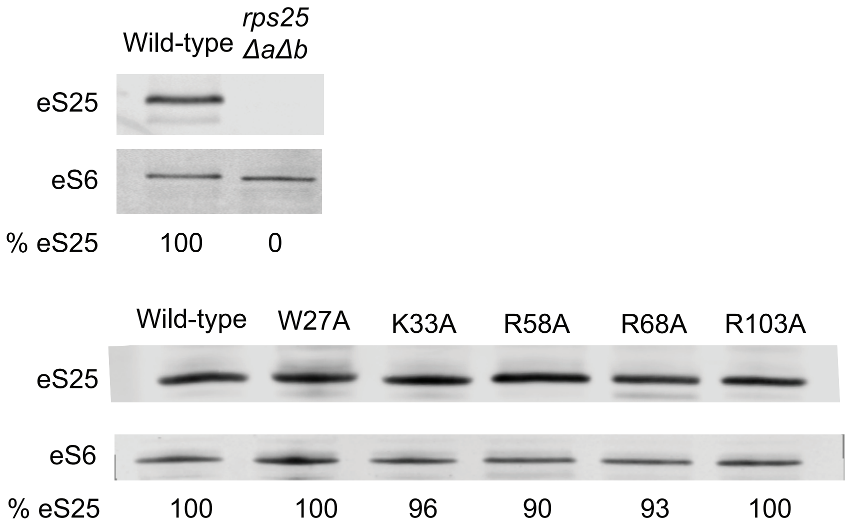

Walters_Supplementary Fig S3

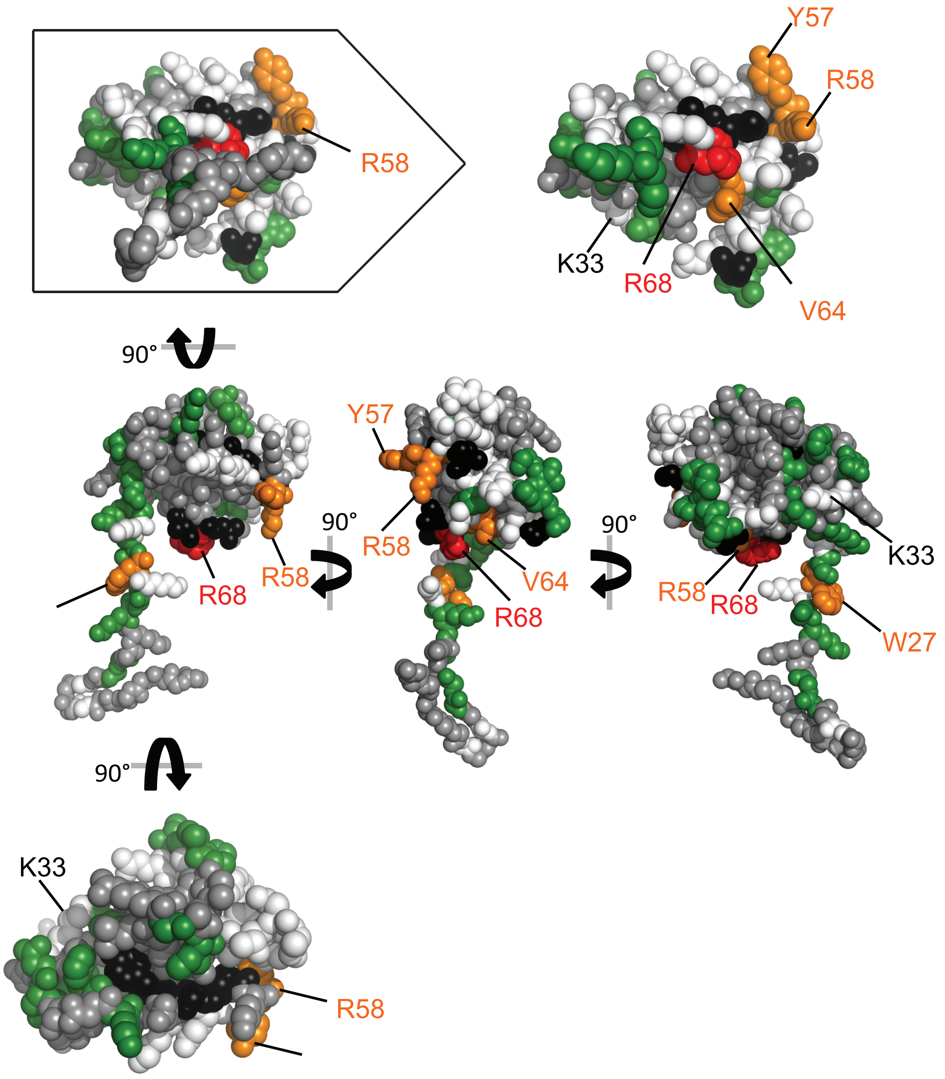

Walters_Supplementary Fig S4

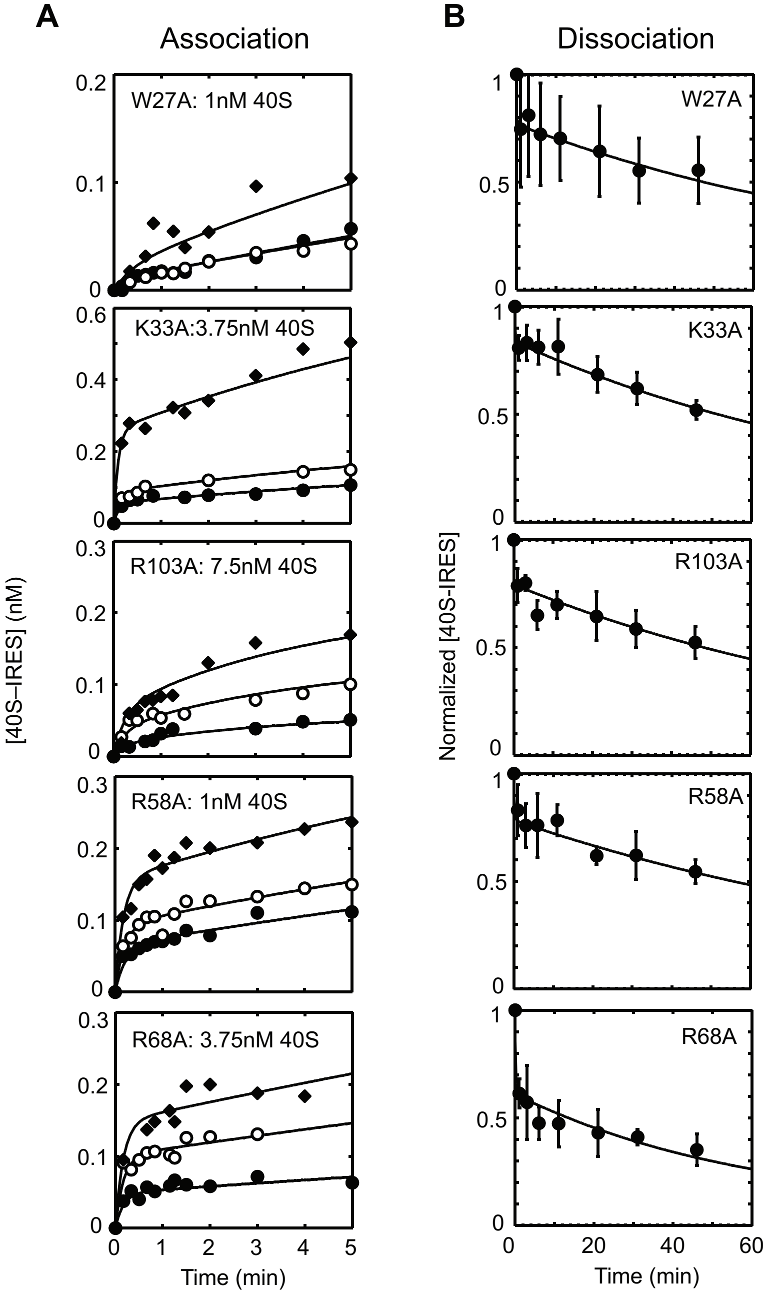

Walters_Supplementary Fig S5

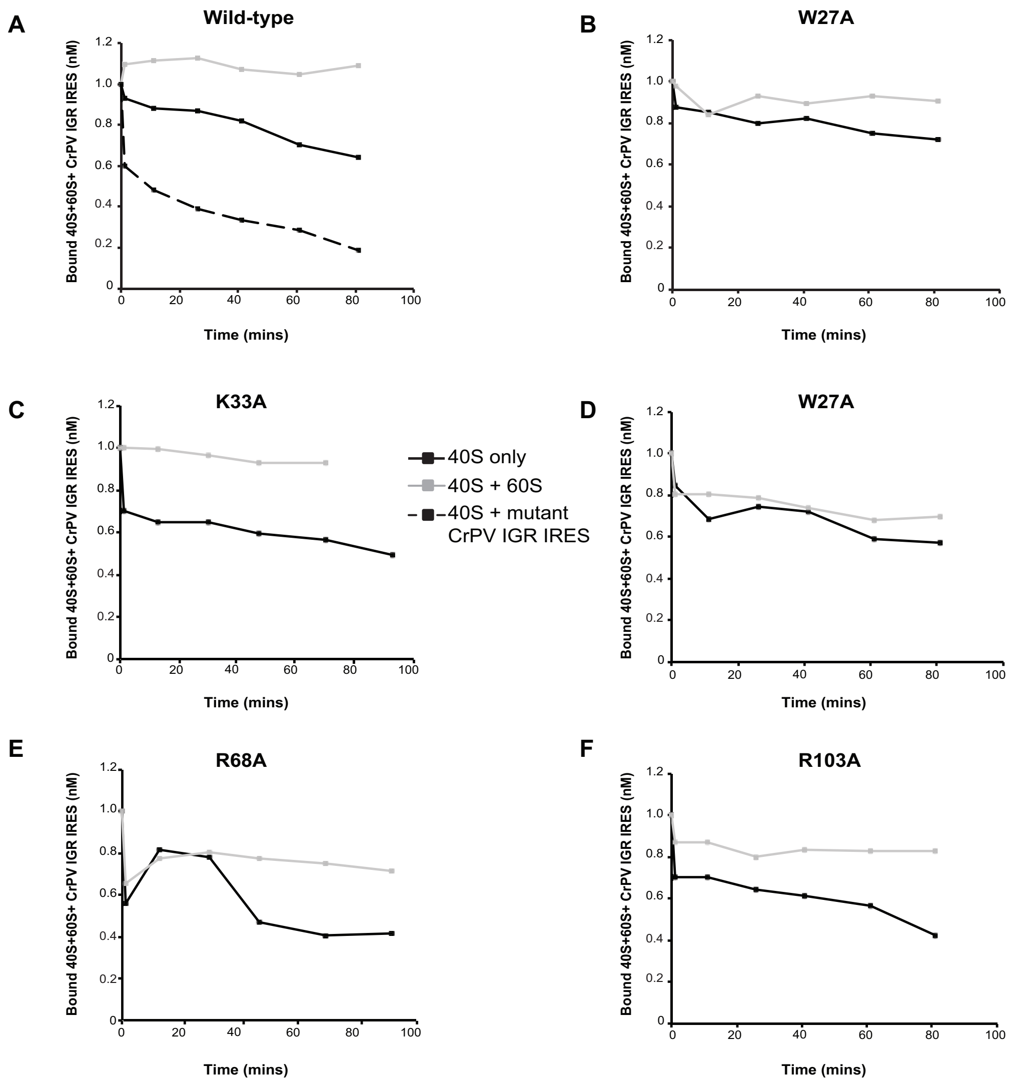

Walters_Supplementary Fig S6

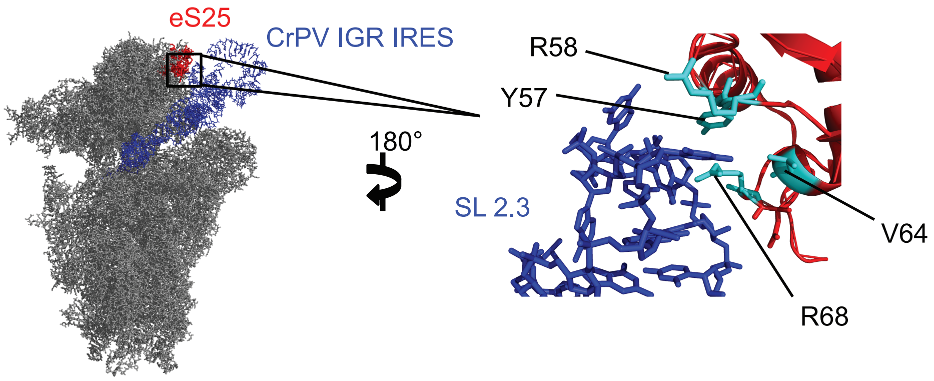

Supplement: gkaa547_Supplemental_File [file gkaa547_supplemental_file.pdf]
